# Physiological Characteristics and Transcriptome Analysis of Exogenous Brassinosteroid-Treated Kiwifruit

**DOI:** 10.3390/ijms242417252

**Published:** 2023-12-08

**Authors:** Chen Chen, Dawei Cheng, Lan Li, Xiaoxu Sun, Shasha He, Ming Li, Jinyong Chen

**Affiliations:** 1 National Key Laboratory for Germplasm Innovation & Utilization of Horticultural Crops, Zhengzhou Fruit Research Institute, Chinese Academy of Agricultural Sciences, Zhengzhou 450009, China; 2 Zhongyuan Research Center, Chinese Academy of Agricultural Sciences, Xinxiang 453514, China

**Keywords:** kiwifruit, NaCl stress, 24-epibrassinolide, physiological conditions, transcriptome

## Abstract

Brassinosteroids (BRs) play pivotal roles in improving plant stress tolerance. To investigate the mechanism of BR regulation of salt tolerance in kiwifruit, we used ‘Hongyang’ kiwifruit as the test material. We exposed the plants to 150 mmol/L NaCl stress and irrigated them with exogenous BR (2,4-epibrassinolide). The phenotypic analysis showed that salt stress significantly inhibited photosynthesis in kiwifruit, leading to a significant increase in the H_2_O_2_ content of leaves and roots and a significant increase in Na^+^/K^+^, resulting in oxidative damage and an ion imbalance. BR treatment resulted in enhanced photosynthesis, reduced H_2_O_2_ content, and reduced Na^+^/K^+^ in leaves, alleviating the salt stress injury. Furthermore, transcriptome enrichment analysis showed that the differentially expressed genes (DEGs) related to BR treatment are involved in pathways such as starch and sucrose metabolism, pentose and glucuronate interconversions, and plant hormone signal transduction, among others. Among the DEGs involved in plant hormone signal transduction, those with the highest expression were involved in abscisic acid signal transduction. Moreover, there was a significant increase in the expression of the *AcHKT1* gene, which regulates ion transduction, and the antioxidant enzyme *AcFSD2* gene, which is a key gene for improving salt tolerance. The data suggest that BRs can improve salt tolerance by regulating ion homeostasis and reducing oxidative stress.

## 1. Introduction

Soil salinization is a growing global problem. According to statistics, the global saline soil area has now reached 1 billion ha [1], and the salinized soil area is increasing annually. When the salt content in the soil exceeds the tolerance range of plants, it produces an excess of harmful substances such as reactive oxygen species (ROS) and causes problems such as decreased photosynthesis, respiratory disorders, and ion imbalances, which seriously affect the growth of plants and reduce agricultural production [2].

*Actinidia* L. is a functional perennial dioecious woody vine genus in the family Actinidiaceae. Kiwifruit is rich in organic matter, such as vitamin C, vitamin E, folic acid, polysaccharides, protein; and trace elements, such as potassium, which are beneficial to the human body [3]. Because of its delicious flavor, it has always been favored by consumers. However, kiwifruit plants are salt-sensitive fruit trees with fleshy roots. When the salt concentration content in the environment is 0.14%, it causes salt damage to the plant [4]. They are suitable for growth in neutral and acidic soils. Salt damage to kiwifruit plants is mainly characterized by a decline in the accumulation of organic matter, an oversupply of required nutrients, inhibition of leaf growth or abscission, shortening of internodes, drying up of branches, and cessation of plant growth or death of the whole plant. However, the problem of soil salinization has become increasingly prominent, and salinity has become an important factor affecting the development of the kiwifruit industry [5,6]; thus, there is an urgent need to improve the salinity tolerance of kiwifruit plants.

In most cases, soil improvement is carried out via measures such as the use of guest soil, trench digging, salt drainage, and water and fertilizer regulation to reduce the salt concentration in the soil, but these methods are time-consuming and laborious. Chemical regulation is an effective approach to improve plant salinity tolerance [7,8,9] to increase the adaptability of plants to salinized land.

Plants have developed systems to cope with salt stress over a long period of evolution, mainly classified into salt avoidance and salt tolerance systems. Salt tolerance mechanisms mainly include regionalization, ion homeostasis, osmoregulation, and antioxidant systems [10]. When subjected to salt stress, plant cells accumulate large amounts of harmful ROS, which can easily lead to lipid peroxidation of cellular membranes and decreased enzyme activity [11]. The major ROS in plants include hydrogen peroxide (H_2_O_2_), superoxide anion (O_2_^•−^), singlet oxygen (^1^O_2_), and the hydroxyl radical (OH^•^) [12]. The antioxidant system is an important enzymatic defense system in plants in which antioxidant enzymes, such as superoxide dismutase (SOD), ascorbate peroxidase, and glutathione reductase, play an important role in preventing the damaging effects of ROS. Osmoregulation is another important mechanism for plants to improve their salt tolerance; increasing the content of osmoregulatory substances in the body increases the osmotic potential of cells and maintains water balance, thus improving salt tolerance [13]. High salt stress causes an ion imbalance in plant cells, resulting in disturbances in ion metabolism, which can lead to increased Na^+^ uptake and decreased K^+^ levels, resulting in Na^+^ poisoning [14]. Once plants perceive salt stress, calcium signals are produced, activating salt-tolerance mechanisms through the SOS (salt overly sensitive) pathway and hormone transport [15].

Brassinosteroids are natural compounds that exist in plants. They have a low concentration in plants but an extremely high physiological activity. BRs are widely involved in various physiological processes in plants [16,17], including the regulation of plant salt tolerance, which has been demonstrated in a variety of plants [18,19,20,21]. A few studies have shown that the exogenous application of BRs can improve salt tolerance in plants, but most of these studies are related to physiology. Few studies have been conducted on kiwifruit. The mechanism by which BRs are involved in increasing salt tolerance in plants is also unclear. Therefore, the present study evaluated the effects of the exogenous application of BRs on salt tolerance by measuring the activity of several relevant physiological indicators in ‘Hongyang’ kiwifruit varieties and determining the appropriate BR concentration that can improve salt tolerance. Combined with RNA sequencing (RNA-Seq), the expression of key genes in response to salt stress was analyzed to better understand the role of BRs in improving salt tolerance in kiwifruit plants.

## 2. Results

### 2.1. Physiological and Biochemical Results

#### 2.1.1. Effect of BRs on the Morphology of Kiwifruit Seedlings under Salt Stress

**Figure 1:**
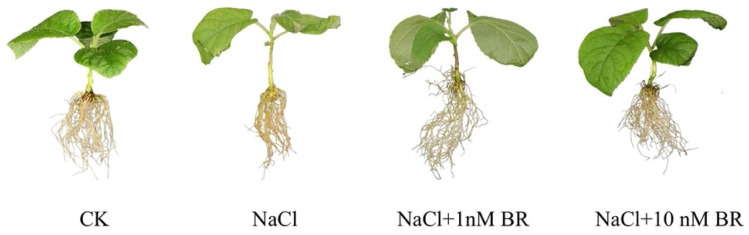
Phenotypes of kiwifruit seedlings treated with NaCl and different brassinosteroid (BR) concentrations.

Kiwifruit seedlings treated only with NaCl showed leaf wilting, yellowing, and curling, drying of the leaf margins, and light brown roots with obvious salt damage. The leaves of plants treated with both BR concentrations showed curling of the leaf margins, and leaves treated with 1 nM BR were more wilted than those treated with 10 nM BR. Overall, the BR-treated plants were slightly damaged after salt stress, and the effect of the BR in alleviating salt stress injury was obvious (Figure 1).

#### 2.1.2. Effect of BRs on Photosynthesis in Kiwifruit Seedlings under Salt Stress

**Figure 2:**
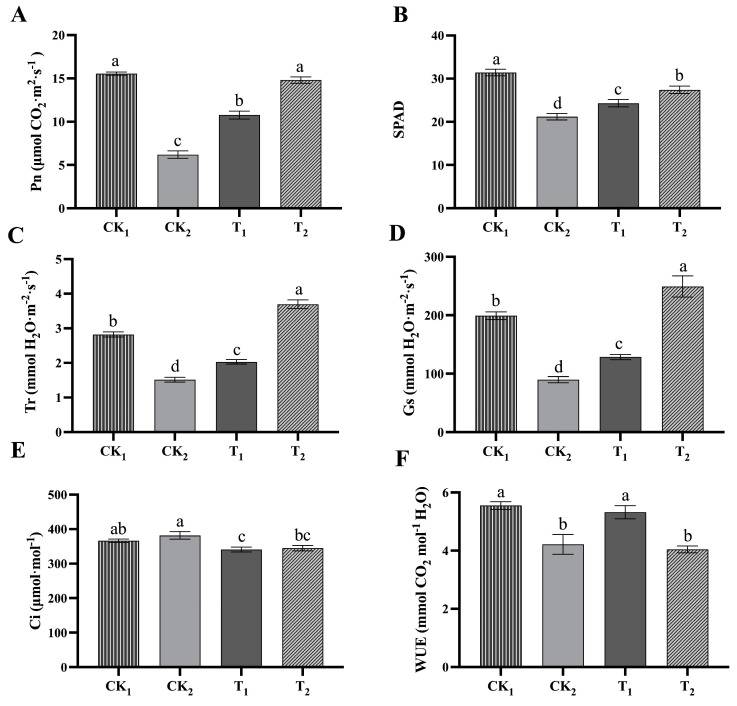
Photosynthetic gas exchange parameters and SPAD values of kiwifruit seedling leaves treated with NaCl and different BR (brassinosteroid) concentrations. (**A**) Net photosynthetic rate (Pn); (**B**) SPAD values; (**C**) Transpiration rate (Tr); (**D**) Stomatal conductance (Gs); (**E**) Intercellular CO_2_ concentration (Ci); (**F**) Photosynthetic water use efficiency (WUE). The data are presented as the mean ± SE (n = 15) from independent experiments. Different lowercase letters indicate significant differences.

Compared to CK_1_, under 150 mmol/L NaCl stress (CK_2_), the Pn, Tr, Gs, WUE, and SPAD values along with the photosynthetic capacity of kiwifruit seedlings decreased significantly, while the Ci value increased. Under NaCl stress, adding 1 nM BR significantly increased the Pn, SPAD, Tr, Gs, and WUE values and significantly reduced the Ci values. Exogenous administration of 10 nM BR resulted in significant increases in the Pn, Tr, Gs, and SPAD values and significant decreases in Ci values, while WUE was not greatly affected (Figure 2).

#### 2.1.3. Effect of BR on Antioxidant Capacity of Kiwifruit Seedlings under Salt Stress

**Figure 3:**
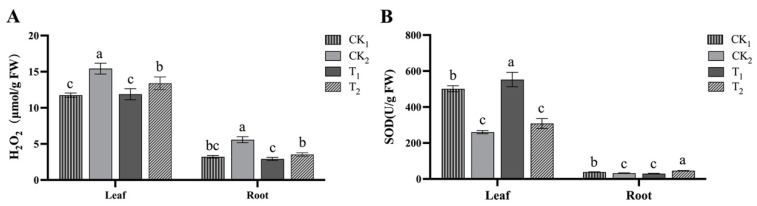
Changes in antioxidant capacity of kiwifruit seedling leaves treated with NaCl and different BR (brassinosteroid) concentrations. (**A**) H_2_O_2_ (hydrogen peroxide) content. (**B**) SOD (superoxide dismutase) activity. The statistical analysis was performed separately for leaves and roots. Three biological replicates were analyzed. The data are presented as the mean ± SD from independent experiments. Different lowercase letters indicate significant differences.

Salt stress caused a significant increase in H_2_O_2_ content in plant leaves and roots, and the BR treatment reduced the H_2_O_2_ content in plant leaves during salt stress. The 1 nM BR treatment reduced the plant H_2_O_2_ content more effectively, with a 23.0% decrease in leaves and a 47.7% decrease in roots (Figure 3A). Salt stress significantly reduced the SOD activity in the leaves and roots of kiwifruit seedlings by 48.1% and 16.0%, respectively. SOD activity increased in leaves after BR treatment, with a significant increase of 112.1% with 1 nM BR, and 42.0% with the 10 nM BR treatment in the roots (Figure 3B).

#### 2.1.4. Effects of BRs on Ion Regulation of Kiwifruit Seedlings under Salt Stress

**Figure 4:**
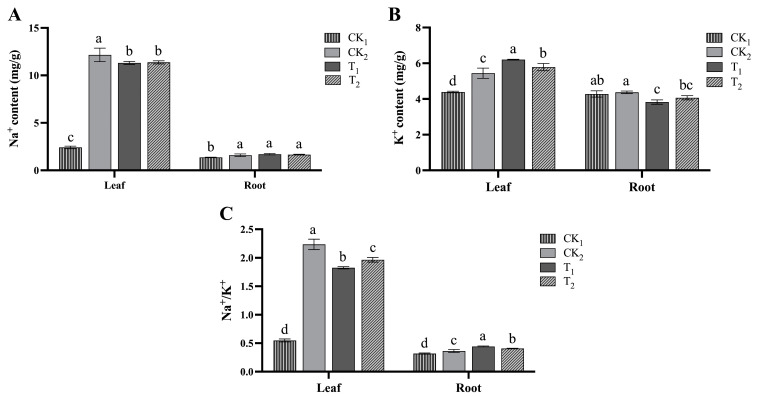
Leaf and root ionic indexes after treatment with NaCl and different BR (brassinosteroid) concentrations. Three biological replicates were analyzed. (**A**) Na^+^ content; (**B**) K^+^ content; (**C**) Na^+^/K^+^ ratio. The statistical analysis was performed separately for leaves and roots. Three biological replicates were analyzed. The data are presented as the mean ± SD from independent experiments. Different lowercase letters indicate significant differences.

Salt stress resulted in a significant increase in the Na^+^ content of leaves and roots. Additionally, both concentrations of BR resulted in a significant decrease in leaf Na^+^ content by 6.93% and 6.49% for the 1 and 10 nM BR treatments, respectively. However, there was a non-significant increase in root Na^+^ content by 6.52% and 4.0% (Figure 4A). The leaf K^+^ content increased significantly under salt stress and increased further with BR treatment: 1 nM and 10 nM BR treatments increased the leaf K^+^ content by 13.9% and 6.28%, respectively. The root K^+^ content increased by 2.15% under salt stress, which was not significant, while it decreased significantly by 12.4% and 6.98% after BR treatment (Figure 4B). In terms of the sodium/potassium ratio, BR treatment resulted in a significant decrease in leaf Na^+^/K^+^ by 18.3% and 12.0% in 1 and 10 nM BR treatments, respectively, and led to a significant increase in Na^+^/K^+^ in the root system by 21.7% and 11.9%, respectively (Figure 4C).

### 2.2. Comprehensive Analysis of Physiological Indicators in Kiwifruit Seedlings

To determine the BR concentration that induces the better salt tolerance, the combined effects of eight dependent variables (Pn, SPAD, L-H_2_O_2_, L-SOD, L-Na^+^/K^+^, R-H_2_O_2_, R-SOD, R-Na^+^/K^+^ (L—leaf; R—root)) were analyzed using principal component analysis. According to the cutoff of an Eigenvalue > 1, three principal components were extracted with a cumulative contribution of 100%, reflecting the overall effect of the different treatments (Table 1). The principal component composite score is the Z-value, and the larger the Z-value, the better the composite effect. The final comprehensive evaluation results were CK_1_ > T_2_ > T_1_ > CK_2_ (Table 2). According to the comprehensive analysis results combined with the overall characterization of the plant and leaf drying, the BR treatments had a mitigating effect on salt stress in the kiwifruit plants, with 10 nM BR being more effective. Therefore, root samples of CK, NaCl, and NaCl + 10 nM BR were sent to Sangon Biotech (Shanghai, China) for transcriptome sequencing.

### 2.3. Transcriptome Results

#### 2.3.1. Sequencing Data Assembly and Mapping

Based on the transcriptome data, nine gene libraries from three treatments with three replicates of CK, NaCl, and NaCl + 10 nM BR were constructed. The sequencing results yielded 39,683,330 to 48,919,526 raw reads (Table 3). Quality cutting using Trimmomatic yielded 37,979,874 to 46,667,374 clean reads, accounting for more than 95% of the raw reads. The sequences after QC were aligned to the reference genome using HISAT2, and more than 83% of the reads were uniquely mapped to the reference genome using the RSeQC statistical alignment results (Table 4). The comparison rate between samples was relatively uniform, indicating that the data were comparable between samples.

#### 2.3.2. Expression of Differential Genes

**Figure 5:**
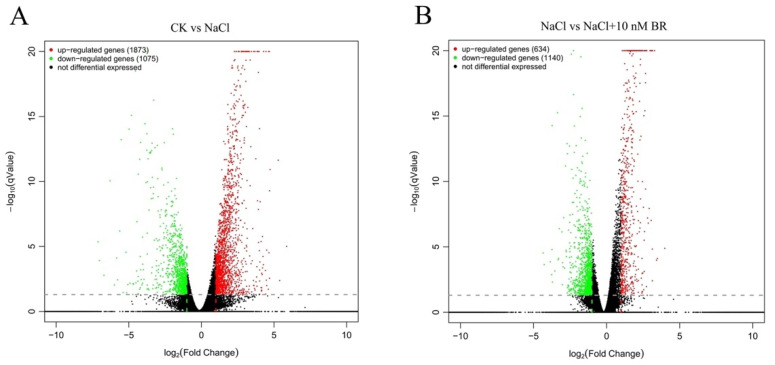
Volcano plot of differentially expressed genes (DEGs) in ‘Hongyang’ root systems, with a fold change > 2 set as the significance threshold. (**A**) CK vs. NaCl; (**B**) NaCl vs. NaCl + 10 nM BR.

**Figure 6:**
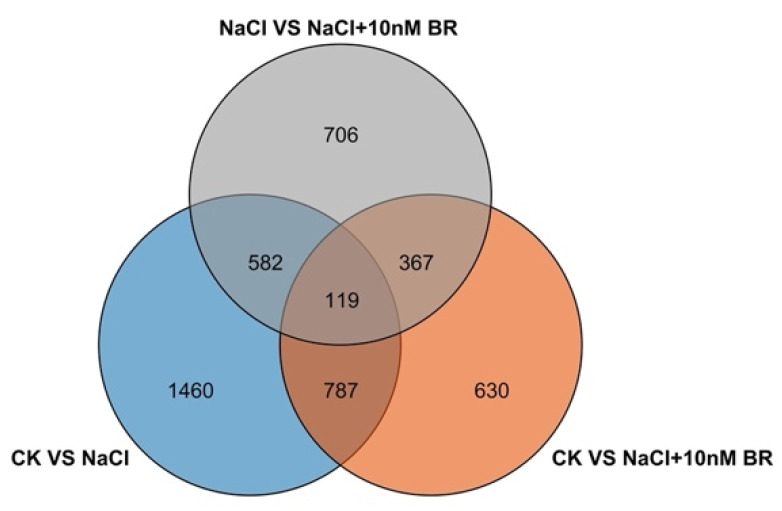
Venn diagram of differentially expressed gene between different treatments treated with salt and a BR (brassinosteroid).

Differentially expressed genes (DEGs) were identified based on differences in gene expression between different samples. The RNA-Seq results showed that 2948 DEGs (1075 downregulated, 1873 upregulated) were identified in CK vs. NaCl (Figure 5A), and 1533 DEGs were enriched and annotated in GO. CK vs. NaCl + 10 nM BR identified 1903 DEGs (1087 downregulated and 816 upregulated), and 997 DEGs were enriched and annotated in GO (Figure 5B). A total of 1774 DEGs (1140 downregulated, 634 upregulated) were identified between NaCl vs. NaCl + 10 nM BR, and 875 DEGs were enriched and annotated in GO, showing that the BR treatment had a significant effect on the transcription of salt stress-related genes. To investigate the relationship between DEGs from the three comparisons, 4651 DEGs were used to construct a Venn diagram. The results suggested that the majority of DEGs in the NaCl and NaCl + BR comparisons may be associated with BR regulation of salt stress. The Venn diagram analyses revealed 119 DEGs shared by the three treatments, representing the core genes associated with the salt stress response (Figure 6).

**Figure 7:**
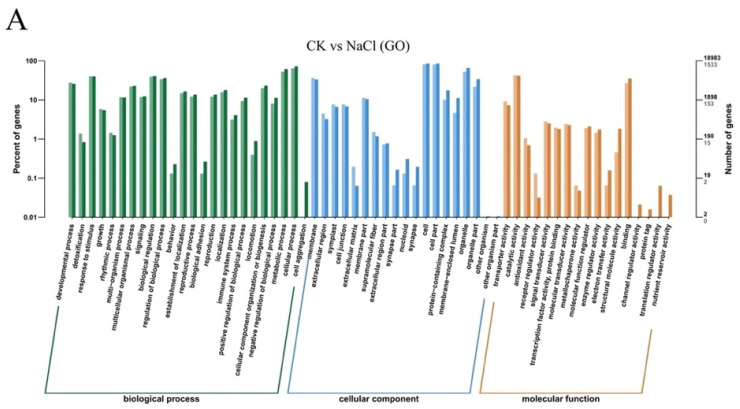

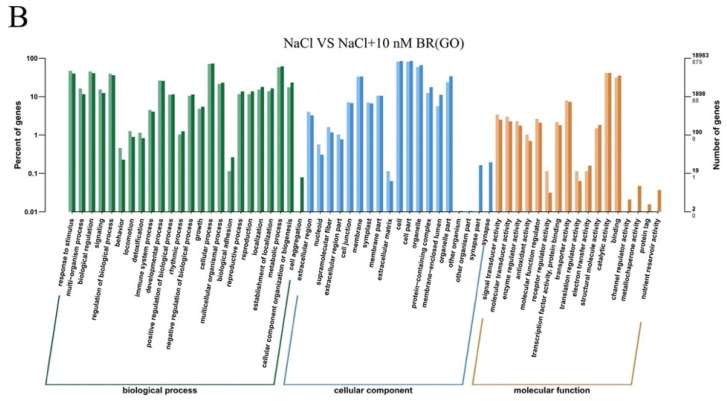
Gene Ontology (GO) enrichment analysis of differentially expressed genes in ‘Hongyang’ root systems. (**A**) CK vs. NaCl; (**B**) NaCl vs. NaCl + 10 nM BR.

The GO classification of the DEGs between the three treatments was similar. In biological processes, the DEGs are mainly involved in cellular processes, metabolic processes, response to stimulus, and biological regulation. In the category of cell components, the differentially expressed genes are mainly related to cells, cell parts, organelles, and membranes. In the category of molecular functions, they are mainly involved in catalytic activity and binding (Figure 7, Table S1). 

To further analyze the metabolic and signal transduction pathways that the DEGs induced by BR treatment participate in, they were annotated using the KEGG public database.

**Figure 8:**
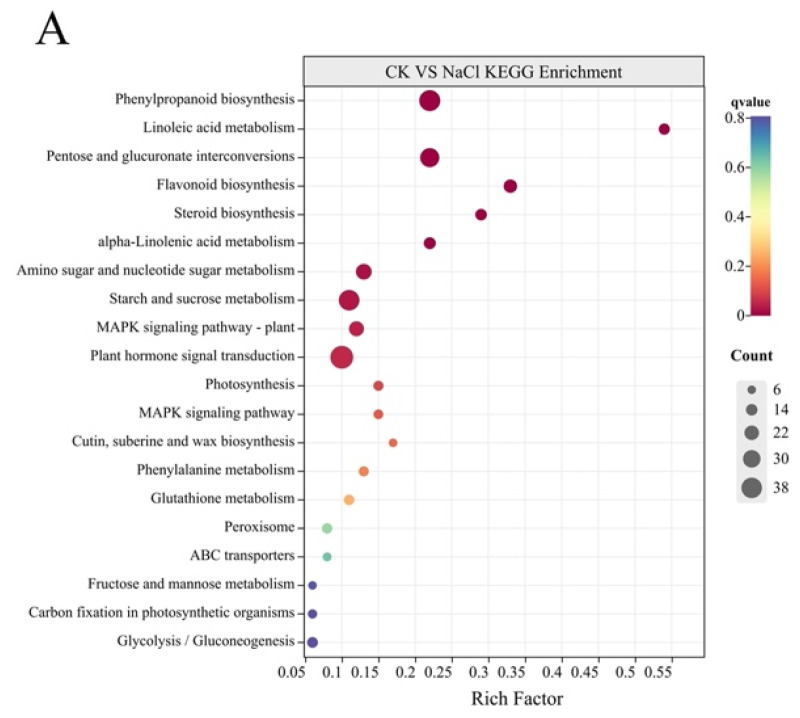

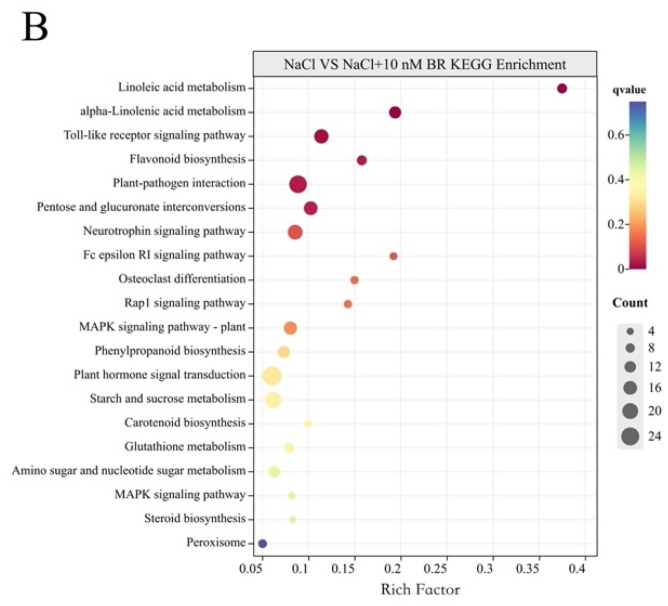
Kyoto Encyclopedia of Genes and Genomes (KEGG) enrichment analysis of differentially expressed genes in ‘Hongyang’ root systems. (**A**) CK vs. NaCl; (**B**) NaCl vs. NaCl + 10 nM BR.

KEGG mapping indicated that the DEGs of CK vs. NaCl were enriched in 236 biological pathways, 31 of which were significantly enriched. The DEGs of NaCl vs. NaCl + 10 nM BR were enriched in 202 biological pathways, among which, six pathways were significantly enriched (*p* < 0.05). In addition, there were high proportions of DEGs involved in biological pathways, such as ‘Plant hormone signal transduction’, ‘Glutathione metabolism’, ‘Phenylpropanoid biosynthesis’, ‘Pentose and glucuronate interconversions’, and ‘Starch and sucrose metabolism’, which may be related to salt tolerance (Figure 8, Table S2).

The main salt stress tolerance pathways in plants are oxidative stress, osmoregulation, and ion homeostasis [10]. Based on the reported mechanisms of salt tolerance in plants and the pathways identified by the KEGG analysis, we focused on the ‘Plant hormone signaling’ pathways and DEGs that regulate ion balance and oxidative stress.

### 2.4. DEGs Induced by BR under Salt Stress

#### 2.4.1. DEGs Related to Plant Hormone Transduction under BR Treatment and Salt Stress

**Figure 9:**
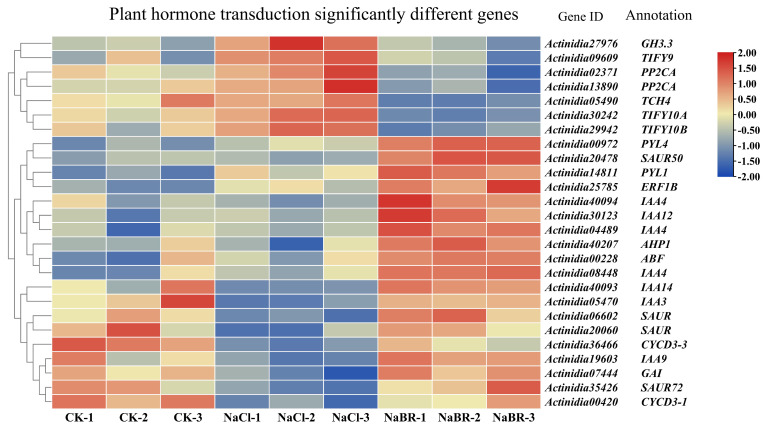
Heatmap of significant differentially expressed genes for plant hormone transduction in brassinosteroid (BR)-treated plants under salt stress.

Comparing the transcriptome data of BR-treated seedlings under salt stress with those under salt treatment alone, 26 DEGs were found to be involved in plant hormone signaling pathways. The DEGs involved in hormone pathways were related to the auxin, cytokinine, gibberellin, abscisic acid, ethylene, brassinosteroid, and jasmonic acid signaling pathways. The genes involved in the auxin and abscisic acid signaling pathways accounted for most of the DEGs. As a result of BR application, 11 out of the 12 DEGs in the auxin signaling pathway were significantly increased, while one was significantly decreased. Additionally, three out of five genes in the abscisic acid signaling pathway were significantly increased and two were significantly decreased (Figure 9).

#### 2.4.2. Differential Genes Involved in BR-Induced Ion Transport under Salt Stress

**Figure 10:**
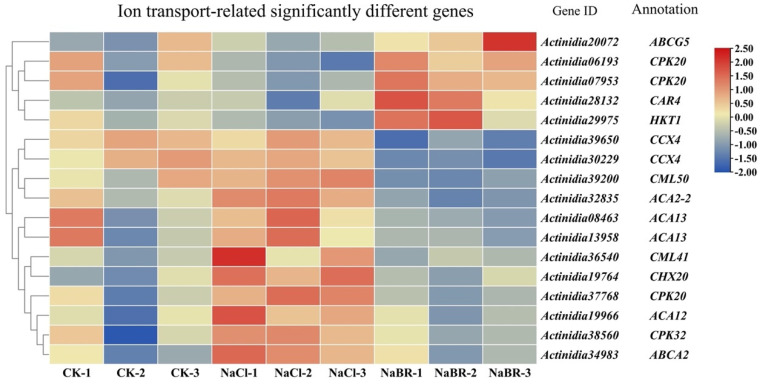
Heatmap of significant differentially expressed genes for calcium, sodium, and potassium ion transport-related genes in brassinosteroid (BR)-treated plants under salt stress.

Combining the KOG and KEGG analysis results, the BR-induced changes in calcium, sodium, and potassium ions that transmit salt stress signals were summarized (Table S3). The BR treatment led to a significant upregulation of four genes compared to the salt treatment alone. According to available reports, two of the *CPK* family genes may encode calcium-dependent protein kinases (CPK), which can act as receptors and effectors of Ca^2+^ for signal transduction [22]. In addition, high-affinity potassium transporters (HKTs) have been reported to have sodium and potassium transporting properties and play an important role in the long-range transport and distribution of sodium and potassium ions in plants [23]. The expression of *HKT1 (AcHKT1)*, an ion transport gene involved in sodium and potassium ion transport that is regulated by salt stress, was significantly increased. This was consistent with the changes in the sodium and potassium ion content in plants after the BR treatment (Figure 10).

#### 2.4.3. Differential Genes for BR-Induced Antioxidants under Salt Stress

**Figure 11:**
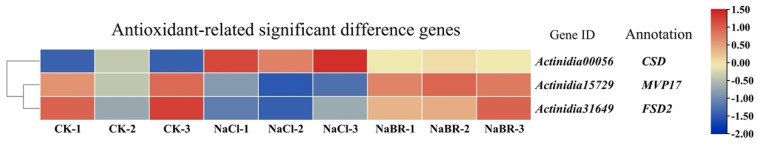
Heatmap of differentially expressed genes for antioxidants in brassinosteroid (BR)-treated plants under salt stress.

Among the DEGs involved in the response to salt stress, we identified three that are associated with oxidative stress and were enriched in the peroxisome pathway, two of which were SOD, which destroys free radicals that are normally produced within cells but are toxic to biological systems. FSD2 plays an important role in chloroplast development, especially in the maintenance of thylakoid membranes [24]. MVP17 is a peroxisome membrane protein. The BR treatment led to a significant increase in *FSD2 (AcFSD2)* and *MVP17 (AcMVP17)* expression (Figure 11, Table S4).

### 2.5. Validation of RNA-Seq Results by qRT-PCR

**Figure 12:**
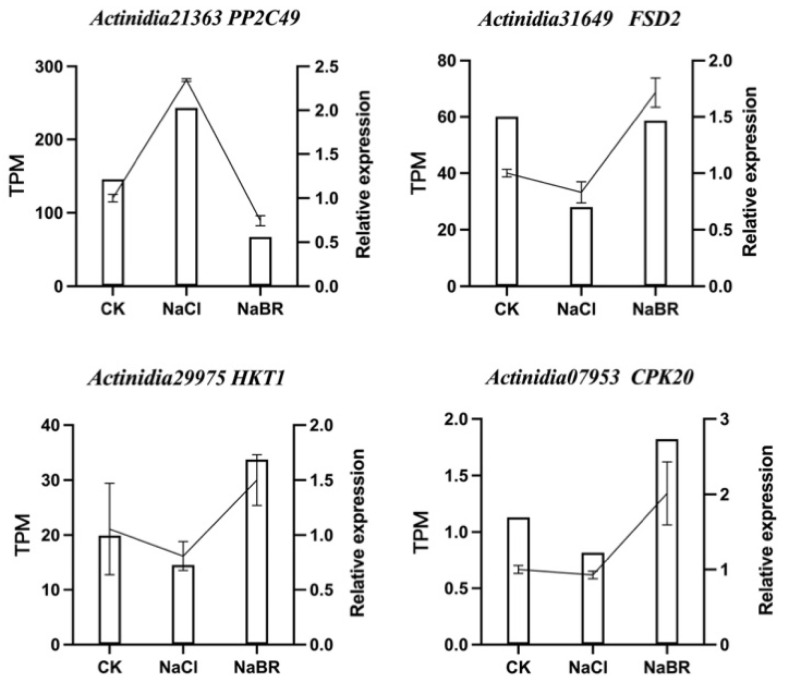
Quantitative RT-PCR analysis of representative genes.

To further validate the RNA-Seq results and assess candidate genes involved in the salt-tolerance pathway, we selected four DEGs. The RT-qPCR data of these genes were consistent with the RNA-Seq results, verifying the reliability of the transcriptome sequencing results (Figure 12, Table S5).

## 3. Discussion

### 3.1. Effects of BR on the Physiology of Kiwifruit Seedlings under Salt Stress

Salt stress causes varying degrees of damage to plant growth and physiological metabolic processes, causing plant death in severe cases [25]. The external morphology and growth status are the most intuitive manifestations of salt stress damage to plants. This study showed that 150 mmol/L salt stress caused serious injury to kiwifruit seedlings. Both 1 and 10 nM BR alleviated oxidative damage and photosynthesis, regulated ionic balance, and improved salt tolerance. Compared with 1 nM BR, 10 nM BR alleviated salt stress more effectively.

Photosynthesis is the main pathway in plant material metabolism and energy conversion, and chlorophyll II is the main pigment in plant photosynthesis and is an important physiological indicator for plant salt tolerance [26,27]. The plant photosynthetic rate, osmotic potential, water potential, and transpiration rate are significantly affected by salt stress [28]. Studies have confirmed that exogenous BR application can restore the photosynthetic efficiency of plants under abiotic stress conditions, such as salt stress, by adjusting the gas exchange parameters [7,8,9,10,11,12,13,14,15,16,17,18,19,20,21,22,23,24,25,26,27,28,29]. Hu et al. reported an increase in the net photosynthetic rate in *Leymus chinensis* [30]. A BR treatment increased Gs and Pn in perennial ryegrass under salt stress (250 mmol/L NaCl) [31]. The decrease in photosynthesis efficiency is the main reason for the inhibition of growth under salt stress, and the factors leading to the decline in photosynthesis are both stomatal factors and non-stomatal factors. The decrease in Ci and Gs is mainly due to the stomatal factors, while the non-stomatal factors are mainly responsible for the increase in Ci and the decrease in Gs [32]. In this study, NaCl stress (150 mmol/L) significantly reduced the Pn, Tr, Gs, WUE, and SPAD values of kiwifruit seedlings, but Ci increased significantly, indicating that the non-stomatal factors (Gs decrease, Ci increase) caused by NaCl stress hindered and reduced the CO_2_ utilization efficiency in the kiwifruit seedlings, thereby causing the Pn and photosynthetic capacity of the kiwifruit seedlings to decrease. This is consistent with previous studies using BRs in oat [33] and eggplant [34]. The application of 10 nM BR under 150 mmol/L NaCl stress resulted in a significant increase in Pn, Tr, Gs, and SPAD values but a significant decrease in Ci (increasing Gs and decreasing Ci), suggesting that exogenous BRs can improve the CO_2_ utilization efficiency of kiwifruit seedlings by maintaining a certain level of stomatal conductance, which, in turn, compensates for the decrease in the photosynthetic capacity of kiwifruit seedlings caused by the NaCl stress. This is consistent with previous results in oat [33], melon [35], tomato [36], and yellow cabbage [37].

The combination of biotic and abiotic stresses exacerbates ROS production, leading to an oxidative burst. Rising levels of antioxidant enzymes help to scavenge excess ROS [38]. SOD constitutes the first line of defense against ROS by converting the superoxide anion to H_2_O_2_ [39]. This study showed that the H_2_O_2_ content of kiwifruit seedlings under salt stress increased significantly, and SOD enzyme activity was significantly inhibited. Different BR concentrations had different degrees of effects on SOD enzyme activity in both leaves and roots. As a result, the H_2_O_2_ content of the seedlings decreased, and the physiological damage was alleviated. This indicates that the appropriate BR concentration can regulate the antioxidant system to cope with stress damage. This is consistent with findings in eggplant [40], *Medicago sativa* [41], and rice [42].

K^+^ and Na^+^ have important physiological functions in the process of plant growth, but these ions can only play normal physiological roles in a relatively balanced state. If the balance is broken, it adversely affects physiological processes [14]. In addition, the ability of plants to maintain the Na^+^/K^+^ ratio under salt stress is closely related to their salt tolerance, and the lower the ratio, the stronger their salt tolerance. Our results showed that the BR treatment increased the K^+^ content under salt stress and could reduce the Na^+^/K^+^ ratio by 15.6% and 6.94% under the 1 and 10 nM BR treatments, respectively. This is consistent with a previous study by Wu et al., who reported that both 10 and 100 nM EBR treatments reduced the Na^+^/K^+^ ratio and alleviated the ion imbalance, suggesting that exogenous BRs can modulate the ion balance and reduce toxicity.

Overall, 150 mmol/L salt stress inhibited photosynthesis in kiwifruit seedlings, resulting in an increased H_2_O_2_ content and increased Na^+^ levels, leading to oxidative damage and an ion imbalance. The application of an exogenous BR, especially at a concentration of 10 nM, increased the net photosynthetic rate of the kiwifruit seedlings, increased the activity of the antioxidant enzyme SOD, increased the K^+^ content in leaves, reduced Na^+^ transport from roots to the aboveground tissues, and reduced the Na^+^/K^+^ ratio. These effects improved the photosynthesis rate, oxidative stress, and ion imbalance in the kiwifruit seedlings, reducing salt stress damage.

### 3.2. Effect of BRs on Differential Gene Expression in Kiwifruit Seedlings under Salt Stress

In the short term, salt stress produces osmotic stress, which reduces plant water absorption and inhibits growth. The long-term harm is mainly due to the toxic effect caused by ion accumulation, especially Na^+^ accumulation, which leads to a Na^+^/K^+^ imbalance and inhibition of photosynthesis, usually accompanied by oxidative stress caused by ROS accumulation. Over a long period of evolution, plants have developed unique ways to cope with salt stress. The molecular mechanisms underlying plant salt tolerance form the basis of physiological mechanisms. The current research on plant salt stress response genes mainly focuses on signal transduction and regulation-related genes, ion transporter genes, and antioxidant-related genes [43].

Ca^2+^ plays an important signal transduction role during salt stress, with Ca^2+^ signals generated by salt stress-induced triggering mechanisms. Four major classes of Ca^2+^-binding proteins and sensors have been identified, including calmodulin (CaM) and similar proteins (CMLs), calcium-dependent protein kinases (CDPKs or CPKs) and their related protein kinases (CRKs), calcineurin B-like proteins (CBLs), and calcium- and calmodulin-dependent protein kinases (CCaMKs) [44]. Among these, calcium-dependent protein kinases (CPKs) are important sensors of calcium signal transduction, showing dual functions as Ca^2+^ responders and sensors [45], and they play a crucial role in the stress response in plants. OsCPK21 positively regulates ABA signaling and salt stress tolerance in rice, and heterologous expression of apple *MdCPK1a* in tobacco improves the tolerance of transgenic plants to salt and cold stress [46,47]. In this experiment, the expression of two *CPK20 (AcCPK20)* genes was significantly increased by the BR treatment, indicating that the BR treatment promoted Ca^2+^ signaling and an active response to salt stress in kiwifruit roots.

The key mechanism for salt tolerance is mainly to reduce the Na^+^ content in plants. There are three main mechanisms known to improve salt tolerance in plants. The first mechanism is excretion through the Na^+^/H^+^ reverse transporter (SOS1) on the plasma membrane, and the second is to transport Na^+^ ions to the vacuole through the Na^+^/H^+^ reverse transporter (AtNHX) on the vacuole membrane, which is thought to be involved in the long-range transport of Na^+^ and the extravasation of roots [48,49]. The high-affinity K^+^ transporter (HKT) is the third transport mechanism. According to its protein structure and ionic selectivity, the HKT type 1 transporter is divided into two subclasses: class 1 members are more selective for Na^+^ than for K^+^, whereas class 2 members are considered to function as Na^+^/K^+^ cotransporters, mediating the balance between Na^+^ and K^+^ ions under salt stress [50]. AtHKT1;1 is the only HKT-type transporter in *Arabidopsis thaliana*. It is predominantly expressed in vascular parenchymal cells around the xylem and localized on the plasma membrane of these cells [50,51,52]. Studies have found that mutations in *AtHKT1;1* inhibit the Na^+^ hypersensitivity of *SOS1* mutants, indicating that AtHKT1;1 is a determinant of plant salt tolerance and controls the Na^+^ balance in plants [53]. Kader et al. found that the transport genes *OsHKT1* and *OsHKT2* in rice were expressed under salt stress, and the Na^+^ concentration may be reduced by adjusting the ratio of Na^+^/K^+^ [54]. Similar results were obtained in this study. The BR treatment increased the expression of *AcHKT1* in kiwifruit plants, reduced the Na^+^ content and Na^+^/K^+^ in the leaves, and improved the salt tolerance of the plants.

Through long-term evolution, plants have developed a series of growth and development mechanisms adapted to high salinity environments. Among these, the hormone regulatory response is one of the core mechanisms in the response to salinity stress [55,56]. BRs regulate salt tolerance in plants by interacting with other plant hormones [57,58]. According to the transcriptome KEGG enrichment analysis, most of the DEGs enriched in the phytohormone signal transduction pathways were related to ABA. ABA is a major hormone involved in the regulation of salinity stress responses, including stomatal closure, ion homeostasis, salt stress-responsive gene expression, and metabolic changes [59,60,61,62]. Studies have shown that BRs interact with ABA to improve salinity tolerance in plants [63,64]. In this process, PYR/PYL receptors sense and bind to intracellular ABA and inactivate PP2C after forming a ternary complex with PP2C phosphatase, thereby activating the downstream targets of PP2C to achieve regulatory effects [65,66]. The PP2C protein phosphatase family is one of the largest gene families in the plant genome. Many *PP2C* gene family members have been reported to be directly involved in plant salt stress regulation [67]. In *Arabidopsis thaliana*, PP2C has A–J branches, and most PP2Cs in group A promote the salt response by mediating the expression of ABA-responsive genes at the transcriptional level. The BR treatment in this study promoted the expression of PYL and PP2C genes in the ABA signaling pathway. Thus, it is speculated that BRs may improve plant salt tolerance by inducing genes in the ABA signaling pathway.

In addition, HKT1 can interact with PP2C49 from the PP2C family to jointly regulate the distribution of systemic Na^+^ during salinity stress. PP2C49 is in the G branch of the PP2C family of genes, and three PP2C genes in this clade are known to be upregulated by salt stress: *PP2C49* (At3G62260), *PP2C50* (At2G25620), and *PP2C51* (At2G33700) [68]. The bioinformatics screen performed by Lan et al. showed that the group G PP2C gene *PP2C49* (AT3G62260) is one of the most sensitive genes to salt stress [69]. *AtPP2CG1* positively modulated salt stress in an ABA-dependent manner, but *PP2C49* acted in an ABA-independent manner in the experiments of Moli Chu et al. In our experiments, the BR treatment resulted in an increase in *AcPP2C49* expression and a concomitant decrease in *AcHKT1* expression, consistent with the findings of Moli Chu et al. PP2C49 negatively regulates AtHKT1;1 activity, thus determining systemic Na^+^ allocation during salt stress.

Salt stress leads to oxidative stress in plant cells. Under normal conditions, the rate of ROS production is low, but when experiencing stress, a short period of exposure causes ROS to accumulate, resulting in injury. Increasing the activity of antioxidant enzymes can strengthen the resistance of plants to stress. SODs rapidly convert superoxide (O_2_^−^) to hydrogen peroxide (H_2_O_2_) and molecular oxygen, being the first line of defense against oxidative damage. Depending on the metal cofactor used, the SODs found in plants are classified into three groups: FeSOD (FSD), MnSOD (MSD), and Cu/Zn-SOD (CSD), which differ in their location within the cell. CSDs are found mainly in mitochondria, chloroplasts, and the cytoplasm. FSDs are mainly found in mitochondria, chloroplasts, and peroxisomes, while MSDs are found in almost all mitochondria and peroxisomes [70]. *MnSOD* expression and activity have been reported to be upregulated under stress conditions [71], and enhanced salt tolerance was found in transgenic *Arabidopsis thaliana* after *MnSOD* overexpression in mitochondria [72]. *CSD* overexpression improves root growth in transgenic plants and enhances salt tolerance in sweet potatoes [73]. *PaSOD* expression positively regulates secondary cell wall synthesis, enhances salt tolerance, and promotes growth in *Arabidopsis thaliana* [74]. Transgenic *Arabidopsis* plants overexpressing *FSD2* and *FSD3* are tolerant to oxidative stress [75]. In this experiment, the BR treatment led to an increase in *AcFSD2* expression, an increase in the content of SOD enzymes, a decrease in the hydrogen peroxide content, and alleviation of oxidative stress. These studies suggest that BR-induced ROS scavenging regulates plant salt acclimation.

**Figure 13:**
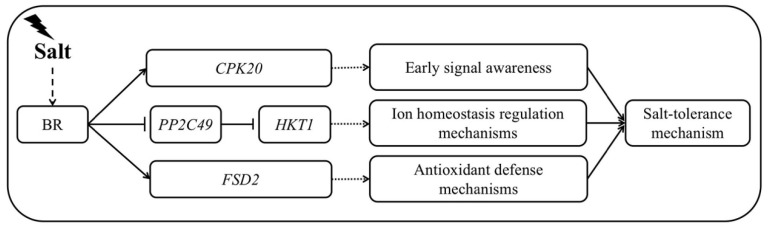
Proposed regulatory network of brassinosteroid (BR) involvement in the salt response.

Based on these results, it is speculated that BR treatment can promote the calcium ion-activated salt stress response. This effect is achieved by increasing the expression of the *AcCPK20* gene and activating the *AcHKT1* response through the inhibition of *AcPP2C49* expression, thereby regulating ion homeostasis and promoting the expression of the antioxidant gene *AcFSD2*. These combined effects improve the antioxidant capacity and the salt tolerance of plants (Figure 13).

## 4. Materials and Methods

### 4.1. Plant Materials and Experiments

The experimental material used was the kiwifruit variety ‘Hongyang’. After 1 week of refining, ‘Hongyang’ kiwifruit seedlings that had been rooted for 45 days were transplanted into nutrient-rich soil (2:1 peat/vermiculite) and placed in a glass greenhouse for one month to continue growing. During this period, Hoagland nutrient solution was applied every 7 days.

The seedlings were assigned to one of four treatments: (i) water (CK_1_), (ii) 150 mmol/L NaCl (CK_2_), (iii) 150 mmol/L NaCl + 1 nM BR (2,4-epibrassinolide, Coolaber; Science & Technology Co., Ltd., Beijing, China) (T_1_), and (iv) 150 mmol/L NaCl + 10 nM BR (T_2_). Kiwifruit seedlings were pretreated with different concentrations of the BR for 24 h before salt treatment. The relevant physiological indexes were determined after 48 h of salt treatment. Each plot contained 15 plants per treatment. Within each treatment, three biological replicates were used.

### 4.2. Measurement of Plant Photosynthesis-Related Parameters

Photosynthetic gas exchange parameters, including photosynthetic rate (Pn), stomatal conductance (Gs), intercellular CO_2_ concentration (Ci), water use efficiency (WUE), and transpiration rate (Tr), were measured in the second fully expanded leaf of the kiwifruit seedlings using a CIRAS-3 Portable Photosynthesis System (PP SYSTEMS, Boston, MA, USA). The test used buffer bottles to supply gas, and the photosynthetically active radiation was set at 1200 μmol m^−2^ s^−1^.

### 4.3. Measurement of SPAD Values

The SPAD (Soil and Plant Analyzer Development) values of the kiwifruit seedlings’ second fully expanded leaf were determined the day before sampling using a SPAD 502DL Plus Chlorophyll Meter (Konica Minolta, Tokyo, Japan).

### 4.4. Measurement of Relevant Biochemical Indicators

The SOD activity and content of H_2_O_2_, Na^+^, and K^+^ were measured using the corresponding assay kits according to the manufacturer’s instructions (Jiangsu Enzyme Immunity Industry Co., Ltd., Yancheng, China). The experiments used three biological replicates.

### 4.5. Statistical Analyses

The data were analyzed using IBM SPSS Statistics 25 (IBM, Inc., Armonk, NY, USA) through one-way analysis of variance (ANOVA), Duncan’s method for ANOVA and multiple comparisons, and principal component analysis for the pooled analysis. LSD was used to determine the significance of differences between treatments (*p* < 0.05) and GraphPad Prism 9 (GraphPad Software, Inc., La Jolla, CA, USA) was used for mapping. Kyoto Encyclopedia of Genes and Genomes (KEGG) enrichment scatter mapping was performed using principal component analysis from https://www.chiplot.online/ (accessed on 5 September 2023). Heat mapping was performed using TBtools-II (V1.120) software.

### 4.6. Transcriptome Sequencing

#### 4.6.1. RNA Extraction and RNA-Seq

Root samples of CK, NaCl, and NaCl + 10 nM BR were sent to Sangon Biotech (Shanghai, China) for transcriptome sequencing. Total RNA was extracted using the Tiangen RNAprep Pure Plant Kit (Tiangen Biotechnology, Beijing, China). After RNA extraction, total RNA was quantified using a Qubit RNA Assay Kit to determine the amount of total RNA added for library construction. The mass and concentration of the RNA samples were measured using an Agilent 2100 bioanalyzer (Agilent Technologies, Santa Clara, CA, USA), a Qubit 3.0 photometer (Invitrogen, Waltham, MA, USA), and a NanoDrop 2000 spectrophotometer (Thermo Fisher Scientific, WA, USA). Magnetic beads containing Oligo(dT) were used for mRNA purification and fragmentation to form short fragments, which were used as templates to construct cDNA libraries. The constructed sample cDNA libraries were sequenced using the Illumina HiSeq4000™ platform (Illumina, San Diego, CA, USA) to obtain the raw data.

#### 4.6.2. Sequence Assembly and Analysis

Quality assessment of the raw data was performed via FastQC, and quality data were trimmed using Trimmomatic to obtain relatively accurate and valid data [76]. Valid data from the samples were compared with the reference genome (‘Hongyang’ V3, http://kiwifruitgenome.org/ (accessed on 10 May 2023)) using HISAT2, and the results were statistically compared using RSeQC [77,78]. Gene expression levels were normalized as Transcripts Per Million (TPM) to analyze gene expression differences between the different samples.

#### 4.6.3. Functional Annotation and Enrichment Analysis of Differentially Expressed Genes

Differential gene expression analysis was performed using DESeq2, with the screening conditions set to q Value < 0.05 and fold difference |FoldChange| > 2. GO enrichment analysis was performed using topGO. KEGG pathway and KOG (EuKaryotic Orthologous Groups) classification enrichment analyses were performed using clusterProfiler.

### 4.7. Quantitative Real-Time PCR (RT-qPCR)

The primers for the target genes were designed using Primer Premier 5 software (Table S6). Reverse transcription was performed using the ReverTra Ace^®^ qPCR RT Kit (Code No. FSQ-101) (TOYOBO, Osaka, Japan). Fluorescence PCR analysis was performed using the NovoStart ^®^SYBR qPCR SuperMix Plus Kit (Novoprotein Scientific Inc., Suzhou, China) and a LightCycler^®^480 instrument (Roche, Fullerton, CA, USA). The PCR amplification conditions were as follows: 95 °C for 5 min, followed by 45 cycles of 10 s at 95 °C, 20 s at 60 °C, and 20 s at 72 °C. Relative expression was calculated using the 2^−∆∆Ct^ method using Aa-actin from kiwifruit plants as a control gene [79,80]. Three independent biological replicates were analyzed.

## 5. Conclusions

Our experimental results show that BR treatment can improve the salt tolerance of kiwifruit plants. In addition, the transcriptome data showed that there may be multiple genes involved in the regulation of salt tolerance by BRs. These results provide new insights into the salt tolerance regulation mechanism of BRs in kiwifruit plants, which is important for understanding and improving the cultivation of salt-tolerant kiwifruit plants.

## Figures and Tables

**Table 1:** Principal component analysis of evaluation factors.

Principal Component	PC1	PC2	PC3
Cumulative variance explained rate (%)	56.865	83.096	100
Weight (%)	56.865	26.231	16.904
Pn	0.195	0.121	−0.286
SPAD	0.205	0.167	−0.051
L-H_2_O_2_	0.2	−0.199	0.024
L-SOD	0.161	−0.303	0.177
R-H_2_O_2_	0.179	−0.25	−0.181
R-SOD	0.071	0.328	−0.481
L-Na^+^/K^+^	0.177	0.159	0.364
R-Na^+^/K^+^	0.074	0.328	0.476

**Table 2:** Comprehensive evaluation of ‘Hongyang’ plants treated with NaCl and different brassinosteroid (BR) concentrations.

	Z Value	Sort
CK (CK_1_)	0.935	1
NaCl (CK_2_)	−0.522	4
NaCl + 1 nM BR (T_1_)	−0.332	3
NaCl + 10 nM BR(T_2_)	−0.081	2

**Table 3:** Total raw and clean reads from salt-treated and BR-treated ‘Hongyang’ root samples.

Sample	Name	Total Raw Reads Count	Total Clean Reads Count	Clean Reads Ratios
Root	CK (CK_1_)-1	46,099,850	44,015,926	95.48%
	CK (CK_1_)-2	62,790,266	59,672,992	95.04%
	CK (CK_1_)-3	37,868,464	36,313,204	95.89%
	NaCl (CK_2_)-1	45,737,256	43,448,892	95.00%
	NaCl (CK_2_)-2	38,979,938	37,349,052	95.82%
	NaCl (CK_2_)-3	43,867,674	41,751,154	95.18%
	NaCl + 10 nM BR(T_2_)-1	40,418,566	38,676,650	95.69%
	NaCl + 10 nM BR(T_2_)-2	39,910,140	38,213,008	95.75%
	NaCl + 10 nM BR(T_2_)-3	38,721,286	37,049,964	95.68%

**Table 4:** Salt-treated and BR-treated ‘Hongyang’ root sample reads were compared with kiwifruit genome database.

Sample	Name	Total Reads	Total Mapped
Root	CK (CK_1_)-1	43,440,582 (100.00%)	37,431,515 (86.17%)
	CK (CK_1_)-2	58,776,918 (100.00%)	50,181,238 (85.38%)
	CK (CK_1_)-3	33,194,052 (100.00%)	28,456,592 (85.73%)
	NaCl (CK_2_)-1	41,902,434 (100.00%)	34,880,169 (83.24%)
	NaCl (CK_2_)-2	36,117,688 (100.00%)	30,721,327 (85.06%)
	NaCl (CK_2_)-3	33,398,428 (100.00%)	28,175,797 (84.36%)
	NaCl + 10 nM BR(T_2_)-1	36,674,346 (100.00%)	30,404,547 (82.90%)
	NaCl + 10 nM BR(T_2_)-2	31,311,032 (100.00%)	26,544,735 (84.78%)
	NaCl + 10 nM BR(T_2_)-3	33,492,586 (100.00%)	27,914,130 (83.34%)

## Data Availability

Due to technical problems, the data is currently being uploaded to NCBI. Additional information will be supplemented after the upload process is complete. Sequence data from this work can be found in the NCBI database (SRA data).

## Supplementary Materials

The following supporting information can be downloaded at: https://www.mdpi.com/article/10.3390/ijms242417252/s1.
